# Meditative Movements for Patients with Type 2 Diabetes: A Systematic Review and Meta-Analysis

**DOI:** 10.1155/2020/5745013

**Published:** 2020-02-01

**Authors:** Tingwei Xia, Yue Yang, Weihong Li, Zhaohui- Tang, Qingsong Huang, Zongrun Li, Yongsong Guo

**Affiliations:** ^1^Chengdu University of TCM, Chengdu, Sichuan Province, China; ^2^Department of TCM, Qingyang District People's Hospital, Chengdu, Sichuan Province, China

## Abstract

**Objective:**

Physical activity plays a specific role in the fundamental aspect of diabetes care. It is necessary to develop exercise programs for these patients. The aim of this systematic review is to summarize current evidence regarding the effectiveness of meditative movement in patients with type 2 diabetes.

**Methods:**

The following databases were searched: PubMed, CENTRAL, Web of Science, Ovid LWW, and EMBASE. Two independent investigators searched and screened the studies by finding duplications, excluding irrelevant titles and abstracts, and then selecting eligible studies by reviewing full texts. 21 studies fulfilled the inclusion criteria. Meta-analyses were performed on glycated hemoglobin (HbA1c), fasting blood glucose (FBG) and postprandial blood glucose (PPBG), total cholesterol (TC), triglycerides (TG), high-density lipoprotein cholesterol (HDL-C), low-density lipoprotein cholesterol (LDL-C), and body mass index (BMI).

**Results:**

Meta-analyses showed that meditative movements significantly improved FBG, HbA1c, PPBG, TC, LDL-C, and HDL-C. No improvement was found in BMI.

**Conclusions:**

The results demonstrated a favorable effect or tendency of meditative movements to improve blood glucose and blood lipid levels in patients with type 2 diabetes mellitus. The special effects of meditative movements in type 2 diabetes mellitus patients need further research.

## 1. Background

Physical activity is an important part of the diabetes lifestyle management and negatively associated with the risk of type 2 diabetes mellitus (T2DM). It plays a specific role in the fundamental aspect of diabetes care [1–3]. Type 2 diabetes is one of the most common diseases in older adults. However, the incidence of children, adolescents, or young people is on the rise, due to the rising level of obesity, lack of physical activity, and poor diet [2]. As the International Diabetes Federation reported, there are approximately 451 million people (ages 18–99 years) with diabetes in the world [3]. And approximately 90–95% of all cases are type 2 diabetes [2]. By 2017, nearly 5 million people between the ages of 20 and 99 had died of diabetes and its complications [4]. At the same time, there are 374 million people with impaired glucose tolerance who are at high risk of developing diabetes [4]. Diabetic complications affect hundreds of millions of patients with type 2 diabetes [5]. T2DM patients have a high risk of liver fibrosis and liver steatosis [6, 7]. Due to the presentation and progression of these complications, patients may lose their vision, kidney, and nerve function. Their activity and cognitive ability may be impaired, and their quality of life may deteriorate. This leads to limited employment and productivity and increased costs for the patient and society [8–11].

Meditative movements, combining breath control, relaxation, musculoskeletal stretching, and a meditative state of mind, have been shown to be effective for treating type 2 diabetes [12]. Meditative movements, including Tai Chi, Yoga, and Qigong, reported by the National Health Interview Survey, are popular among American adults in workplace [13]. Yoga and Tai Chi, especially, are recommended by the American Diabetes Association for older adults with type 2 diabetes to increase flexibility, muscular strength, and balance [1]. Plenty of clinical researches have focused on the effectiveness of meditative movements on type 2 diabetes. Present systematic reviews or meta-analyses about meditative movements have shown that it is beneficial to chronic obstructive pulmonary disease, sleep quality, cancer, and major depressive disorder [14–17].

However, the systematic review and meta-analysis of meditative movements on type 2 diabetes have not been conducted. Therefore, we performed a systematic review and meta-analysis to evaluate the effectiveness of meditative movements as a complementary therapy for patients with type 2 diabetes.

## 2. Data and Methods

This review was performed according to our previous protocol [18]. Our protocol of this systematic review and meta-analysis on PROSPERO was registered in advance (no. CRD42019128495, https://www.crd.york.ac.uk/PROSPERO).

### 2.1. Data Sources and Search Strategies

The following databases were searched using the developed search strategy [18] from inception to December 2018: PubMed, Cochrane Central Register of Controlled Trials (CENTRAL), Web of Science, Ovid LWW, and EMBASE.

### 2.2. Inclusion and Exclusion Criteria

We identified studies using the following inclusion criteria as in our protocol [18]: participants (with a clear diagnosis of type 2 diabetes), intervention (Tai Chi or Qigong or Yoga), control (any type of control group), primary outcomes (HbA1c, FBG, and PPBG), secondary outcomes (TC, TG, HDL-C, LDL-C, and BMI), and study type (randomized controlled trials (RCTs)).

### 2.3. Trials Inclusion and Data Extraction

Two investigators independently searched and screened the studies. The process of study selection was performed using the methods according to the PRISMA guidelines [19]. Data extraction was performed by two investigators independently. Data extraction contained, in addition to outcomes, information regarding country of origin, number of randomized participants, number of participants included in type of intervention, frequency of intervention, and duration of intervention. Finally, all differences were resolved by consensus.

### 2.4. Trials Quality Assessment

Definitions in the assessment of bias risk of a trial were conducted according to the Cochrane Handbook criteria for judging the ROB with the “risk of bias” assessment tool [20]. The following domains should be evaluated: random sequence generation, allocation concealment, blinding of participants and investigators, the blindness of outcome assessments, incomplete outcome data, selective outcome reporting, and other biases. The quality of studies was divided into three categories: low, unclear, or high bias.

### 2.5. Statistical Analysis

We used the RevMan 5.3.0 provided by Cochrane Collaboration to analyze the results of the studies. This meta-analysis only included continuous data, so we expressed them as the mean ± standard deviation and then calculated the standardized mean difference (SMD) and obtained the two-sided *P* value and 95% confidence interval (CI). The complete case data was used as the analysis data. The degree of heterogeneity was quantified using the *χ*^2^ test and *I*^2^ value. We performed subgroup analysis according to total sample size (>60 versus ≤ 60), duration (>3 months versus ≤ 3 months), control type (nonexercise versus other active exercises), type of meditative movement (Tai Chi/Qigong versus Yoga), and region (Asia versus non-Asia), as well as a sensitivity analysis if necessary. A test for the interaction between the treatment and subgroups was performed to examine whether treatment effects differed among subgroups. An interaction of *P* value ≥0.05 was considered to indicate that the effect of treatment did not differ significantly among subgroups. Publication bias was assessed by visual inspection of a funnel plot.

## 3. Results

### 3.1. Literature Screening

**Figure 1:**
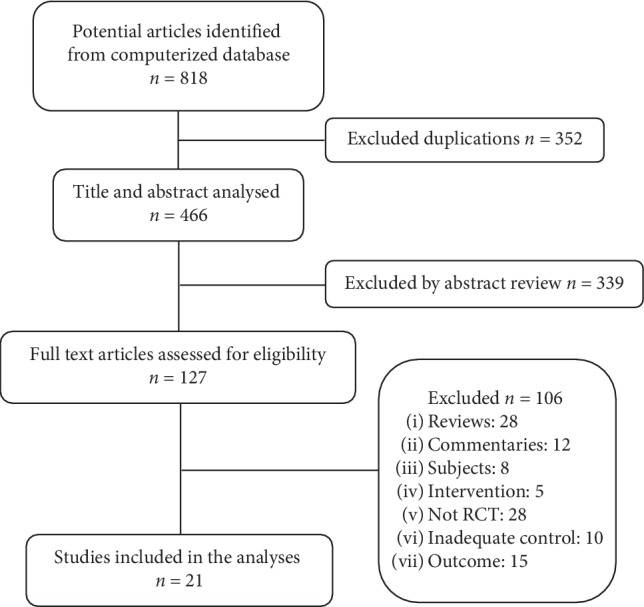
Flow chart of selection process.

We retrieved 818 original papers from the electronic bibliographic databases. The full text of 127 articles was assessed according to the predetermined inclusion criteria. Finally, 21 studies fulfilled the inclusion criteria and were further analyzed [21–44]. The detailed process of the studies evaluation and the reasons for exclusion are shown in Figure 1.

### 3.2. Characteristics of Included Studies

The characteristics of these included trials are described in Table 1. Among them, the data of three studies were reported by six different articles [24–27, 37, 38]. Patients included in these studies were from China [23, 39], Taiwan (China) [29], India [22, 24–27, 31–35], Iran [41], Japan [42], Thailand [30], Australia [40, 43, 44], Cuba [37, 38], and USA [21, 28, 36]. Six studies offered Tai Chi [23, 29, 30, 39, 40, 44], three studies offered Qigong [28, 42, 43], and twelve studies offered Yoga [21, 22, 24–27, 31–38, 41]. The sample sizes of the included studies ranged from 10 to 277. The treatment duration lasted from 45 days to 36 weeks. The frequency ranged from 2 to 7 times weekly, and exercise time lasted 10–120 min per session. Controls were divided into nonexercise groups and other active exercise groups. The exercise forms of other active exercise groups include seated calisthenics, stretching, aerobic exercise plus home-based exercise, progressive resistance training, and physical activity. In three studies, two control groups were set up in each, including nonexercise and other active exercises [28, 31, 37, 38].

### 3.3. Risk of Bias of Studies

**Figure 2:**
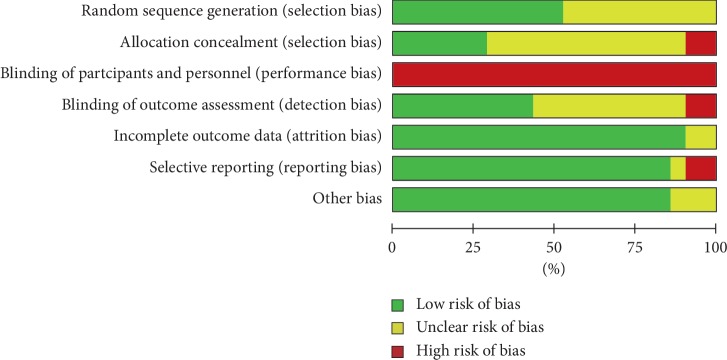
Risk of bias graph.

**Figure 3:**
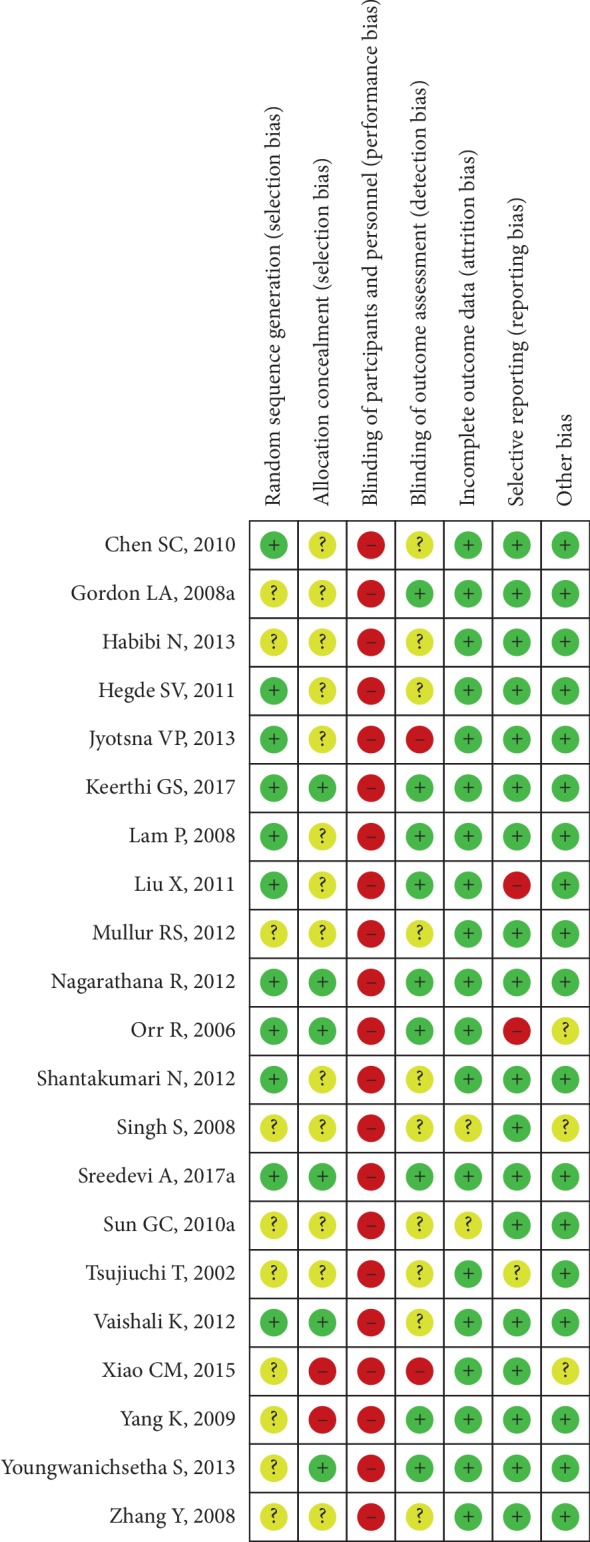
Risk of bias summary.

The bias condition of selected studies was shown in Figures 2 and 3. We assessed the risk of bias in all included articles. Eleven studies used the generation of the allocation sequence. Allocation concealment was used in 6 studies. None of the studies blinded their participants. However, nine studies were blinding of outcome assessors to the treatment allocation, whereas the risk of selective reporting bias was not reported in most studies.

## 4. Outcome

### 4.1. Glycemic Control

**Figure 4:**
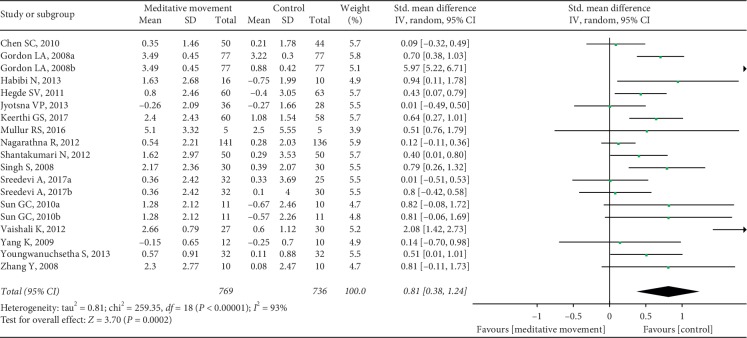
Forest plot of the comparison between meditation movements and the control group for the outcome FBG.

Sixteen RCTs, with three studies setting up two control groups in each, reported FBG as a primary outcome. We split the study with two control groups into two sets of data for summary analysis. A total of 19 sets of data were included. The combined result was statistically significant (SMD = 0.81, 95% CI (0.38, 1.24), *P*=0.0002) compared to the control group, with high heterogeneity (*I*^2^ = 93%, *P* < 0.00001) (Figure 4). We carried out sensitivity analyses to explore potential sources of heterogeneity, and the results did not change substantively. The heterogeneity ranged from 67% to 93%. So, we conducted subgroup analyses and interaction tests according to the total sample size, duration, control type, intervention type, and region. Test for interaction showed significant results between subgroups of the nonexercise and other active exercises (*P*-interaction = 0.002). The result indicated that the difference of the control types was partly the reason why there was severe heterogeneity in the overall analysis. The detailed results are shown in Table 2.

**Figure 5:**
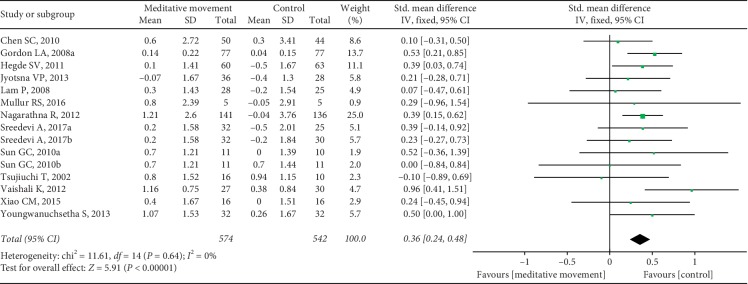
Forest plot of the comparison between meditation movements and the control group for the outcome HbA1c.

**Figure 6:**
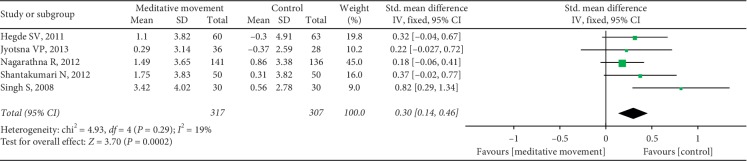
Forest plot of the comparison between meditation movements and the control group for the outcome PPBG.

Thirteen studies reported HbA1c. Sixteen sets of data were included. The heterogeneity was high (*P* < 0.00001, *I*^2^ = 92%). We carried out sensitivity analyses to investigate the potential sources of heterogeneity. After removing one set of data, the results changed obviously. The heterogeneity was calculated as *P*=0.64, *I*^2^ = 0%. The combined result was statistically significant (SMD = 0.36, 95% CI (0.24, 0.48), and *P* < 0.00001) (Figure 5). It showed that one study was the potential source of heterogeneity. However, when we looked up the study again, we did not find differences in methodology and other aspects. The study showed that meditation movements had more significant effects on HbA1c than other studies. Five studies reported the PPBG. It showed a favorable effect of meditation movements on reducing PPBG (SMD = 0.30, 95% CI (0.14, 0.46), and *P*=0.0002), with low heterogeneity (*P*=0.29, *I*^2^ = 19%) (Figure 6).

### 4.2. Lipid Profile

**Figure 7:**
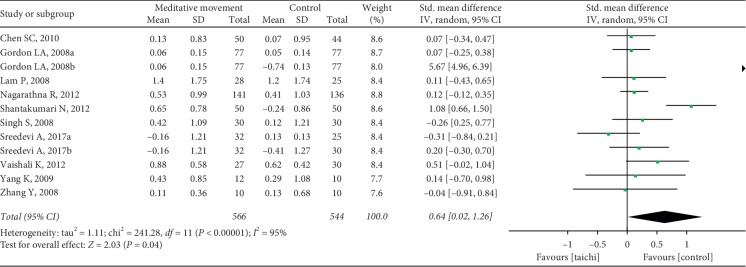
Forest plot of the comparison between meditation movements and the control group for the outcome TC.

**Figure 8:**
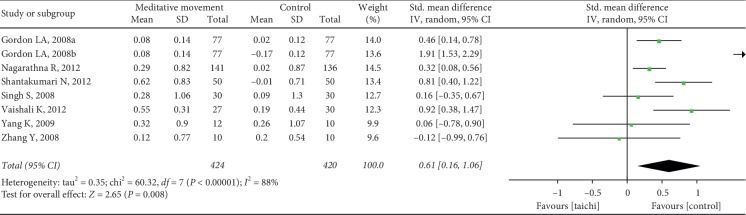
Forest plot of the comparison between meditation movements and the control group for the outcome LDL.

**Figure 9:**
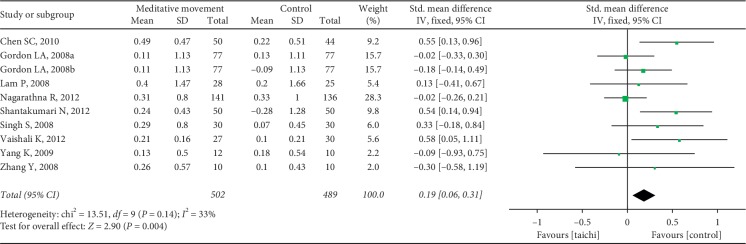
Forest plot of the comparison between meditation movements and the control group for the outcome TG.

**Figure 10:**
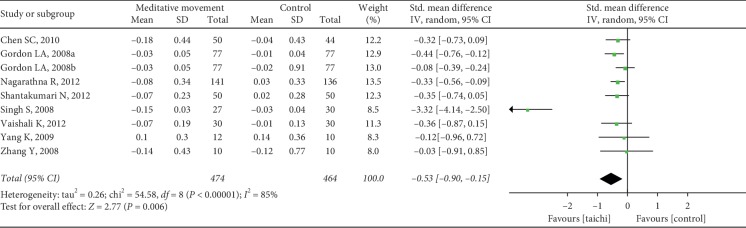
Forest plot of the comparison between meditation movements and the control group for the outcome HDL.

The aggregated results suggested that the meditation movements had significant effects on TC (SMD = 0.64, 95% CI (0.02, 1.26), and *P*=0.04; *P* for heterogeneity < 0.00001, I^2^ = 95%) (Figure 7), LDL-C (SMD = 0.61, 95% CI (0.16, 1.06), and *P*=0.008; *P* for heterogeneity < 0.00001, *I*^2^ = 88%) (Figure 8), triglycerides (SMD = 0.19, 95% CI (0.06, 0.31), and *P*=0.004; P for heterogeneity = 0.14, *I*^2^ = 33%) (Figure 9), and HDL-C (SMD = −0.53, 95% CI (−0.90, −0.15), and *P*=0.006; *P* for heterogeneity < 0.00001, I^2^ = 85%) (Figure 10).

**Figure 11:**
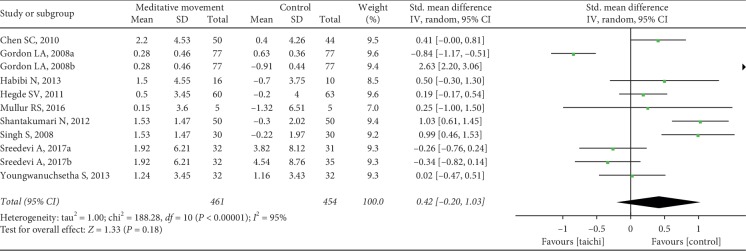
Forest plot of the comparison between meditation movements and the control group for the outcome BMI.

It showed no effects of meditation movements on reducing BMI (SMD = 0.42, 95% CI (−0.20, 1.03), and *P*=0.18) with low heterogeneity (*P* < 0.00001, *I*^2^ = 95%) (Figure 11).

### 4.3. Publication Bias

**Figure 12:**
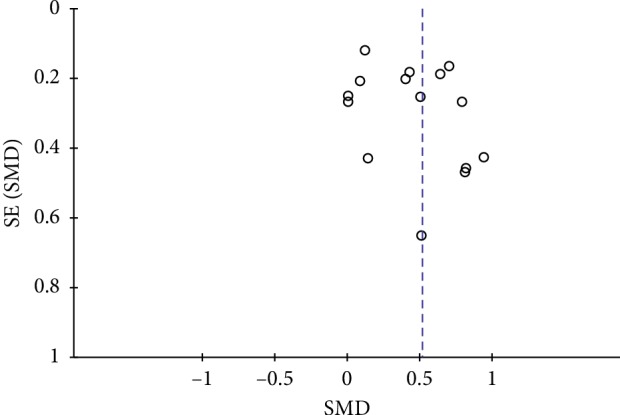
Evaluation of publication bias for FBG.

The FBG included in the study was selected as an indicator. It can be seen that the graph is not obviously asymmetrical (Figure 12). There might have been no publication bias in the comparison of meditation movements and the control group.

## 5. Discussion

Meditative movements (specifically Tai Chi, Qigong, and Yoga), including a focus of the mind on the body and breathing for deep relaxation, are special forms of exercise. More and more studies have been conducted on the effectiveness of these practices in health and healing [14]. As the first systematic review and meta-analysis synthesize the evidence of the effects of meditative movements on type 2 diabetes, we found that meditative movements may have positive effects on the treatment of type 2 diabetes. This evidence suggests that there is a possibility for using these exercises as an augmentation approach to control blood glucose for type 2 diabetes.

### 5.1. Summary of Main Results

The present results showed that the meditative movements significantly improved FBG, HbA1c, PPBG, TC, LDL-C, and HDL-C in patients with type 2 diabetes mellitus. No improvement was found in BMI.

As for the primary outcomes, significant heterogeneity was noted during our analyses of FBG and HbA1c. Sensitivity analyses were carried out to explore the potential sources of heterogeneity for FBG. We found that the heterogeneity or the synthesized results of studies on FBG did not change substantively. Therefore, subgroup analyses and interaction tests were carried out to investigate the impact of various exclusion criteria according to the total sample size, duration, control type, intervention type, and region. No evidence of heterogeneity was observed within the total sample size, duration, intervention type, and region. However, the overall combined effects of the trials showed significant results between subgroups of the nonexercise and other active exercises. It indicated that the reason for heterogeneity might be caused by the difference of the control types. Although the results showed a significant difference in reducing FBG between meditative movements and other active exercises, it was more significant compared to the nonexercise group. There is no doubt that other active exercises had a better effect on lowering blood sugar than nonexercise. Sensitivity subgroup analyses were also conducted to explore the potential sources of heterogeneity for HbA1c. We found only one study was the potential source of heterogeneity where no differences were found in methodology and other aspects. It showed more significant effects of meditation movements on HbA1c than other studies.

Psychological stress has been proven to play a role in the etiology of type 2 diabetes [45]. It is regarded both as a predictor of new-onset type 2 diabetes and as a prognostic factor in people with existing type 2 diabetes. The disturbances across multiple biological systems reflecting chronic allostatic load might exist [46]. Numerous studies have shown that it is a common independent risk factor for disease occurrence [47–50]. Meditative movements could be regarded as a combination of mindfulness intervention and physical activity [51]. This characteristic determines that its intervention in type 2 diabetes is multifaceted. Diaphragmatic breathing practice might be beneficial to reduce negative subjective and physiological consequences of stress in healthy adults [52]. This might partly explain why meditative movements have a more positive influence on type 2 diabetes, comparing to other active exercises and nonexercises. Yoga and Tai Chi are mainly recommended to increase flexibility, muscle strength, and balance, which shows that the particularity of meditative movements is not yet well-known. Plenty of studies have shown that meditative movements are effective for glucose control in patients with type 2 diabetes [53–56]. It is necessary to develop exercise programs because the optimal form of exercise and appropriate parameters of exercise in type 2 diabetes patients are not yet clear.

### 5.2. Limitations

Several limitations have to be mentioned. Heterogeneity among the studies was significant. We conducted sensitivity analyses and subgroup analyses. The control types, other active exercises and nonexercises, might be the main source of heterogeneity. To some extent, they could explain the source of heterogeneity. But the risk of bias and heterogeneity could also be caused by study quality or the exercise intensity. Because participants cannot be blinded to the meditative movements, performance bias could not be ruled out. The distribution of the included studies is also a great concern. Most trials were conducted in Asia or America. No studies were from the European countries. Due to the limited number of included studies in Qigong, more comprehensive subgroups could not be made. This may have influenced the explanatory effect and the soundness of the pooled effects. Since we only performed a search for English studies, it is possible that articles may have been published in other languages.

### 5.3. Implications for Research

There are a few points that should be considered in the future. The methodological quality of these studies was poor in random sequence generation, allocation concealment, and blinding of outcome assessment. More studies with rigorous design and normative description are needed in this field. We first summarized the current condition of meditative movements for type 2 diabetes. The particularity of meditative movements, which differs from purely physical activity, should be valued in future studies.

In summary, based on the evidence, meditative movements have significant effects on controlling blood glucose and blood lipid levels in patients with type 2 diabetes mellitus. These results support the idea that meditative movements are a possible alternative exercise for type 2 diabetes mellitus management. Due to the aforementioned limitations and potential bias, more high-quality randomized controlled studies should be conducted. In addition to increasing flexibility, muscle strength, and balance, the special effects of meditative movements in type 2 diabetes mellitus patients still need further research.

## Figures and Tables

**Table 1:** Characteristics of included studies.

Authors, year		Location	Participants	Number	Experimental group			Control group	Follow-up	Outcome measures
					Types of treatment	Duration (min)	Frequency			
Zhang Y., 2008		China	Women with T2DM	E: 10; C: 10	Tai Chi	60 min	Five times weekly	Nonexercise	14 weeks	(1)FBG (2)TC, HDL-C, LDL-C, TG
Chen S. C., 2010		Taiwan (China)	Patients with T2DM	E: 56; C: 48	Tai Chi	60 min	Three times weekly	Other active exercises (aerobic exercise plus home-based exercise)	12 weeks	(1)HbA1c, FBG
Lam P., 2008		Australia	Adults with T2DM	E: 28; C: 25	Tai Chi	60 min	Two classes weekly	Nonexercise	24 weeks	(1)HbA1c (2)TC, TG
Youngwanichsetha S., 2013		Thailand	Women with T2DM	E:32; CG:32	Tai Chi	50 min	Three times weekly	Nonexercise	12 weeks	(1)HbA1c, FBG (2) BMI
ORR R., 2006		Australia	Older adults with T2DM	E: 17; C: 18	Tai Chi	60 min	Twice weekly	Other active exercises (sham exercise, e.g., seated calisthenics and stretching)	16 weeks	(1) FBG
Xiao C. M., 2015		China	Older adults with DM	E: 16; C: 16	Tai Chi	1 to 2 hours	Three sessions per week	Nonexercise	12 weeks	(1)HbA1c
Sreedevi A., 2017		India	Women with diabetes	E:41; C1:42; C2:41	Yoga	60 min	Twice weekly	C1: other active exercises (physical activity); C2: nonexercise	12 weeks	(1)HbA1c, FBG (2) TC, BMI
Hegde S. V., 2011		India	People with type 2 diabetes	E:60; C:63	Yoga	n.r.	Three times weekly	Nonexercise	12 weeks	(1)HbA1c, FBG, PPBG (2) BMI
Keerthi G. S., 2017		India	People with type 2 diabetes	E:62; C:62	Yoga	45 min	Three times weekly	Nonexercise	12 weeks	(1) FBG
Gordon L. A., 2008 and Gordon L.,2008		Cuba	People with type 2 diabetes	E:77; C1:77; C2:77	Yoga	60 min	3–4 times per week	C1: other active exercises (conventional physical training); C2: Nonexercise	24 weeks	(1)HbA1c, FBG (2)TG, TC, HDL-C, LDL-C, BMI
Singh S., 2008 and Kyizom T., 2010		India	People with type 2 diabetes	E:30; C:30	Yoga	45 min	5 days per week	Nonexercise	45 days	(1)FBG, PPBG (2)TG, TC, HDL-C, LDL-C, BMI
Mullur R. S., 2016		USA	People with type 2 diabetes	E:5; C:5	Yoga	10 min	n.r.	Nonexercise	12 weeks	(1)HbA1c, FBG (2)BMI
Yang K., 2009		USA	People with type 2 diabetes	E:13; C:10	Yoga	60 min	Twice per week	Nonexercise	12 weeks	(1)FBG (2)TG, TC, HDL-C, LDL-C
Jyotsna V. P., 2013		India	People with type 2 diabetes	E:36; C:28	Yoga	n.r.	7 days per week	Other active exercises (brisk walking)	24 weeks	(1)HbA1c, FBG, PPBG
Nagarathna R., 2012		India	People with type 2 diabetes	E:141; C:136	Yoga	60 min	5–7 days per week	Other active exercises (physical exercises)	36 weeks	(1)HbA1c, FBG, PPBG (2)TG, TC, HDL-C, LDL-C
Habibi N., 2013		Iran	Women with T2DM	E:16; C:10	Yoga	75 min	Three times weekly	n.r.	12 weeks	(1)FBG (2)BMI
Shantakumari N., 2012 and Shantakumari N., 2013		India	People with type 2 diabetes	E:50; C:50	Yoga	60 min	7 days per week	Nonexercise	12 weeks	(1)FBG, PPBG (2)TG, TC, HDL-C, LDL-C, BMI
Vaishali K., 2012		India	People with type 2 diabetes	E:27; C:30	Yoga	45–60 min	6 days per week	Nonexercise	12 weeks	(1)HbA1c, FBG (2)TC, TG, HDL-C, LDL-C
Liu X., 2011	Australia	People with type 2 diabetes	E:20; C:21	Qigong	1–1.5 hour	Three times weekly	Nonexercise	12 weeks	(1)HbA1c, FBG, PPBG (2)HDL-C, TG
Sun G. C., 2010	USA	Adults with type 2 diabetes	E:11; C1:10; C2:11	Qigong	30–60 min	Three times weekly	C1: nonexercise; C2: Other active exercises (the progressive resistance training)	12 weeks	(1)HbA1c, FBG;
Tsujiuchi T., 2002	Japan	Adults with type 2 diabetes	E:16; C:10	Qigong	2 h	n.r.	Nonexercise	16 weeks	(1)HbA1c

E: experimental; C: control; FBG: fasting blood glucose; HbA1c: glycated hemoglobin; TC: total cholesterol; TG: triglycerides; HDL-C: high-density lipoprotein cholesterol; LDL-C: low-density lipoprotein cholesterol; BMI: body mass index; n.r.: not reported.

**Table 2:** Subgroup analyses based on various exclusion criteria for FBG.

Subgroup	*n*	SMDs, mmol/L (95% CI)	*I* ^2^ (%)	Heterogeneity, *P* value	*P* interaction
Total sample size					0.87
>60	10	0.84 (0.22, 1.47)	96	<0.00001	
≤60	9	0.91 (0.38, 1.44)	76	<0.0001	
Duration					0.11
>3 months	5	1.79 (0.30, 3.28)	98	<0.00001	
≤3 months	14	0.55 (0.29, 0.80)	65	0.0004	
Control type					0.002
Nonexercise	12	1.42 (0.71, 2.13)	93	0.64	
Other active exercises	6	0.27 (0.15, 0.38)	0	<0.00001	
Intervention type					0.71
Tai Chi/Qigong	5	0.74 (0.16, 1.32)	72	0.007	
Yoga	14	0.89 (0.35, 1.44)	95	<0.00001	
Region					0.28
Asia	13	1.50 (-0.25, 3.25)	78	<0.00001	
Non-Asia	6	0.53 (0.25, 0.80)	97	<0.00001	

CIs, confidence intervals; n, number of trials; SMDs, standardized mean differences.
